# Meeting of the Faculties of the Dental Colleges of the United States

**Published:** 1884-09

**Authors:** E. G. Betty


### MEETING OF THE FACULTIES OF THE DENTAL COLLEGES OF THE UNITED STATES.

Reported for The Independent Practitioner by E. G. Betty, D. D. S.

The conference of Dental College Faculties met at the Sturtevant House, in New York City, at 10 a. m., Monday, Aug. 4, 1884.

Prof. Foster announced the object of the meeting, and moved that Prof. C. N. Pierce act as temporary chairman.

This motion prevailed, and Prof. Pierce took the chair.

Prof. H. A. Smith was appointed to act as temporary Secretary.

Prof. Winder moved that an association be formed, and in pursuance of this a committee of three was appointed on organization, viz: Professors Truman, Taft and Winder.

A motion to admit reporters was carried.

The list of names and representatives of colleges was called for, and responses made as follows:

Baltimore Dental College, Profs. Winder and Foster.
University of Michigan, Prof. J. Taft.
University of Pennsylvania, Prof. Truman.
Philadelphia College, Prof. Garrettson.
Harvard University, Prof. Fillebrown.
Ohio College, Prof. H. A. Smith.
Boston College, Profs. Blodgett and Follett.
Chicago College, Profs. Harlan and Gardiner.
Iowa College, Prof. A. O. Hunt.
Pennsylvania College, Profs. Pierce and Leffmann.
New York College, Prof. Abbott.
University of California, } Represented by letter.
Kansas City College, }

The Committee on Organization was allowed thirty minutes to report, during which the meeting took a recess.

The committee then made the following report:

CONSTITUTION.

SECTION 1. This body shall be called the "National Association of Dental Faculties."

SEC. 2. The objects of this association shall be to promote the interests of dental education.

SEC. 3. The officers shall consist of a President, a Vice-President, a Secretary and a Treasurer, who shall be elected by ballot, and shall hold office till their successors are elected.

SEC. 4. Two-thirds of the colleges belonging to this association shall be necessary to constitute a quorum.

SEC. 5. Any contemplated change, involving the interest of the schools represented in the association, shall require one year's notice before any action is taken.

SEC. 6. Two members shall be elected, who, with the officers, shall constitute an Executive Committee, that shall have power to designate the time and place of the meeting, and make preparation for the same, and pass upon all candidates.

SEC. 7. Any reputable dental college may be represented in this body, upon submitting to the Executive Committee satisfactory credentials, signing the constitution, conforming to the rules and regulations of this body, and paying such assessments as may be made.

*Prof. Winder*—Moved that the report be read, section by section, so that it could be passed upon. Carried.

The first, second and third sections were adopted as read. There was some discussion by Prof. Blodgett as to how each college should vote, whether as a unit or not; it was determined that two-thirds of the representatives should constitute a quorum, each college voting as a unit. Upon this the fourth section was adopted.

In the fifth section, after some remarks pro and con, the section was changed to read "dental schools represented in this body" as above. The section was then adopted as amended.

Section six was changed to read *three* members to be elected instead of *two*. Adopted as amended.

The seventh section, on resolution of Prof. Harlan, was amended to include a membership fee; which resolution was amended on motion of Prof. Taft, to collect funds by assessment *pro rata*. The section was adopted as amended.

Some one moved that a Membership Committee be appointed.

*Prof. Foster*—Said the Executive Committee had power, and was sufficient to perform this duty.

The report, as a whole, was then adopted as such.

The following colleges then signed the constitution:

Baltimore College of Dental Surgery, M. W. Foster, R. B. Winder.

Pennsylvania College of Dental Surgery, C. N. Pierce, Henry Leffmann.

Philadelphia Dental College, J. E. Garrettson.

New York College of Dentistry, Frank Abbott, J. Bond Littig.

Boston Dental College, J. A. Follett, Albert N. Blodgett.

Dental College of the University of Michigan, J. Taft.

University of Iowa, Dental Department, A. O. Hunt.

Chicago College of Dental Surgery, A. W. Harlan, Frank H. Gardiner.

Dental Department of the University of Pennsylvania, James Truman.

Ohio College of Dental Surgery, H. A. Smith.

*Prof. Fillebrown*—Could not sign, as he had no authority to act for his college, but expressed hearty sympathy with the movement.

*Prof. Taft*—Moved that during the intervals between meetings, any college could become a member by applying to the Executive Committee, and presenting satisfactory credentials, and conforming to the rules and regulations as provided in the constitution. Carried.

The association then proceeded to the election of permanent officers, to serve the coming term, with this result:

President, C. N. Pierce; Vice-President, R. B. Winder; Secretary, H. A. Smith; Treasurer, A. W. Harlan.

The three members of the Executive Committee elected were Profs. Abbott (chairman), Truman and Taft.

*Prof. Winder*—Offered a resolution that after the session of 1884–85, the colleges announce in their catalogues that five years' practice will not be an equivalent for one course of lectures.

*Prof. Taft*—Said it was necessary to designate just how many years are required, as the resolution on this point is indefinite.

*Prof. Winder*—Said that his resolution was one of inquiry merely.

*Prof. Truman*—Moved that two full years' attendance at college be required, instead of Prof. Winder's resolution. (Applause.) Carried.

*Prof. Smith*—Made a motion to the effect that graduates of medicine be required to attend but one course in a dental college, provided he pass a satisfactory examination.

*Prof. Abbott*—Moved to amend by requiring such medical graduate to attend one *full year* in college instead of but one course, so that he may have some practical experience; the year to include the college course of lectures.

*Prof. Foster*—Thought the student's ability should determine the length of time.

*Prof. Taft*—Said that reference should be had to men of average ability, and not to the extremes as mentioned. He had had most trouble with medical graduates, because they presume upon their attainments, and do not, in consequence, work and study as they ought. The best way is to require time enough to study thoroughly.

*Prof. Smith*—Said the resolution is indefinite. Why not say "in the college infirmary"?

*Prof. Abbott*—Accepted this, and the resolution as amended was carried

Prof. Smith's resolution, as amended by Prof. Abbott, was carried unanimously.

Prof. Abbott's resolution, recognizing one course of medicine as equivalent to one year's office pupilage, but requiring of such student the attendance upon two courses of dental lectures, was carried.

*Prof. Harlan*—Offered this paragraph as an addition: "Provided that a candidate for graduation shall have attended two regular courses of lectures in separate years." Carried unanimously.

*Prof. Taft*—Offered the following resolution:

*Resolved*, That we recommend that three years' study of dentistry be required for graduation from a dental college.

*Prof. Follett*—Moved to amend by substituting "require" instead of "recommend." There being no second, the amendment fell. The original resolution was then carried.

A motion of Prof. Taft to appoint a committee of three to consider curriculum, was carried. Profs. Taft, Truman and Winder were appointed.

Adjourned.

AFTERNOON SESSION.

Prof. Pierce taking the chair, business was resumed.

*Prof. Winder*—Thought it was best for the association to meet bi-annually.

*Prof. Taft*—Said it was best to meet next year, in order to perfect the business of this meeting.

Prof. Taft's amendment was carried.

He also moved to assess each college the sum of two dollars, to defray the necessary expenses incurred. Carried.

Bills amounting to $15.25 were ordered paid.

*Prof. Truman*—Moved a reconsideration of the action taken, requiring medical graduation as a substitute for one year's course.

*Profs. Winder and Taft*—Were opposed to this, and on a vote being taken, the motion was lost.

*Prof. Garrettson*—Offered the following resolution:

*Resolved*, That the colleges of this association receive into the senior class only such juniors as hold certificates of having passed a satisfactory examination in the studies of the junior year; this certificate to be a pledge to any college to which they may apply, that a previous term has been properly spent in the institution whence they come.

After considerable discussion on this resolution it was referred to the Committee on Curriculum, to be brought up at Saratoga, August 5.

Adjourned.

SARATOGA, Aug. 5, 1884.

The meeting was called to order by President Pierce at 5 P. M.

The minutes of the previous meeting were adopted, with the corrections made.

*Prof. Taft*—Read a letter from the Dean of the Dental Department of the University of California.

*Prof. Abbott*—Said that a college cannot vote by proxy, as colleges must be present and sign the constitution personally by their representatives, and that the letter or proxy cannot constitute membership.

The Chair stated that the constitution provided for colleges to join in the interim, but not by proxy.

*Prof. Truman*—Asked if a college could not vote by proxy, after it had signed.

The Chair said that no provision had been made, and that further legislation must be had upon that point before such action could be taken.

*Prof. Taft*—Moved that Prof. Truman be allowed to act as proxy for Philadelphia.

The Chair stated that this could be done by common consent.

No objection was offered.

*Prof. Smith*—Said that Prof. Garrettson's resolution was laid on the table.

*Prof. Gardiner*—Said it was laid on the table before the morning adjournment, and was brought up again in the afternoon, and referred to the Committee on Curriculum.

The Chair said it could be brought up as new business.

A motion to this effect prevailed, when Prof. Abbott moved that it be referred to the Committee on Curriculum. Carried.

*Prof. Taft*—Desired its adoption, while Prof. Truman favored its reference to the committee.

Prof. Taft, Chairman of the Committee on Curriculum, made the following report:

PRELIMINARY EXAMINATION.

Your committee recommend that a preliminary examination be required for entrance to our dental colleges. Such requirements shall include a good English education.

In case of any student failing to pass a satisfactory preliminary examination, the other colleges of this association may be informed of the fact.

*Prof. Abbott*—Moved that the report be accepted. Carried.

He also said that he thought the report contemplated action a year hence, and in view of that would move that it be adopted.

*Prof. Truman*—Said the latter part, regarding a graded course, was a recommendation, and he could not see how some schools could change their present schedule to comply with it, and expressed his doubt about its practicability.

*Prof. Darby*—Said that the association could recommend the adoption to the schools, and, at any rate, it is but a transposition of studies, which does not seem to be impossible.

*Prof. Foster*—Said that there was a misunderstanding, as it was not intended to prevent its operation the first year. He thought it best that the Deans be notified, so that students could not go from college to college, as it is evident that one college is sufficient to determine his ability.

*Prof. Hunt*—Said that it is usual in institutions to accept literary degrees without examination.

*Prof. Gardiner*—Wanted to know if the preliminary examination was to be in writing.

The Chair said that it was not so stated.

*Prof. Gardiner*—Remarked that it ought to be.

*Prof. Truman*—Said that in his judgment the examination should be a test of general information.

*Prof. Taft*—In order to test the sentiment of the meeting, suggested that the resolution be amended to read "a reasonable knowledge of numbers, geography and history."

*Prof. Foster*—Said that was just the point where we split; we stopped just at "the English language." In some branches, as Prof. Taft suggested, some candidates are deficient, though they may be generally intelligent. The examining board ought to be the best judges of proficiency.

*Prof. Abbott*—Said that the examination ought to be in writing, and that a simple statement of a preliminary examination is enough, without specifying what the examination is to consist of.

*Prof. Taft*—Said that all he wanted was that all candidates be, at least, as well educated as school-boys of ten years of age. We ought, at least, to expect a little more than this.

*Prof. Foster*—Said that an ordinary English education comprised the studies mentioned.

*Prof. Taft*—Said that he meant an ordinary English education, as generally understood.

*Prof. Foster*—Made a motion to amend, so that it would read "ordinary English education."

*Prof. Follett*—Said the candidate ought to advance a little on such an education; he ought to know numbers, geography and history, but the "history" ought to be limited, as the term is very broad.

*Prof. Taft*—Said that it is modified by the word "reasonable" in the report.

*Prof. Follett*—Asked that in case a candidate failed to pass examination, would another college refuse him, and is it justice to the student?

*Prof. Smith*—Said that he may come back after a time and pass a satisfactory examination; better not do the student injustice, as he may be satisfactory to one college, and not another.

*Prof. Follett*—Thought that the clause must mean something; say, let the student study one year further, and then try again for his examination.

*Prof. Foster*—The object of the report is, that when applying to one college the student is found not competent, the other Deans be notified. A Dean's notification is sufficient evidence of the student's incompetency at that time, for, as he could not enter one college, neither could he any other, at that time.

*Prof. Follett*—Expressed the hope that the examination be, at least, partly in writing.

*Prof. Darby*—Said that it is possible for a student to go rapidly from one college to another, and represent himself as never having been examined, so that it is possible for us to make mistakes, because one Dean may not notify others immediately.

*Prof. Foster*—The examination may or may not be severe, and if one college refuses a student another one may accept him, and the onus is thrown upon it, while the other gets the honor, or, at least, assumes it.

*Prof. Abbott*—Said that certain days could be set apart for the preliminary examination, so as to avoid this trouble.

*Prof. Truman*—There is a practical difficulty about this matter. The Dean often has as much as he can do from Sept. 15th to Oct. 15th in receiving students, without making examinations; these would delay matters till away into October, and what will be done with the students in the meanwhile?

*Prof. Smith*—Said that we are attempting too much at this meeting; we are not ready to specify examinations. It would be better to wait until we learn what the Medical College Association has been doing. It is better to go slowly, and not adopt faulty methods.

*Prof. Gardiner*—Said that in a certain institution named, the college examines and keeps the papers on file just the same as in the final examination, so that they are open for inspection at any time.

*Prof. Follett*—What I want to know is, that if I refuse to receive a rejected man, will the other colleges do the same for mine that I reject? Otherwise, what is the use of notification?

*Prof. Tuft*—I don't apprehend any trouble from this cause, as, in all probability, there will not be more than two or three cases of this kind in a year, and hardly that many. When the action of the College Association is known, students will prepare themselves before applying. Six or eight hours is sufficient to examine forty or fifty student's with paper and pencil.

*Prof. Smith*—Requested the Chair to give an opinion upon the subject.

*Prof. Pierce*—Said that he had been interested in examining medical students for beneficiaries, in which the application of the student is in writing. They may send credentials from teachers in whose school they were engaged, then the pastor certifies to general character. These examinations number about twelve in a year. It is an impossibility to formulate certain questions, or name certain days for examination. The admission can, in many cases, be conducted by correspondence.

*Prof. Smith*—Said that, in his opinion, a graduate of a literary institution needs no examination, and that it is an insult to put it upon gentlemen of such attainments.

*Prof. Taft*—Diplomas of this kind are always accepted when coming from respectable institutions. The report may be changed to embrace this point, but it was understood.

*Prof. Smith*—Moved to amend, by inserting the clause "carried by common consent."

The first part of the report as amended was carried unanimously.

*Prof. Taft*—Then read the second section of the report.

*Prof. Truman*—Moved to adjourn, that they might think about the matter over night.

*Prof. Foster*—Did not think it sufficiently important for that.

*Prof. Smith*—Said he did not want to presume, but why not strike out "specification" and insert "graded course."

Adjourned to meet Aug. 6, at 4 30 P. M.

August 6, 1884.

The Chair called the meeting to order.

The reading of the minutes was dispensed with.

*Prof. Abbott*—Offered the following resolution:

*Resolved*, That we agree to adopt a graded course of instruction, and an intermediate examination, which course of instruction and examination shall be conducted as the faculties of the different colleges represented in this association may deem proper.

*Prof. Truman*—Moved its adoption.

*Prof. Abbott*—On being asked, said that he offered it as a substitute for that portion which refers to graded course, etc.

The resolution was carried unanimously.

*Prof. Hunt*—Wanted to get some information as to the manner of issuing certificates, and if there was any change contemplated in the manner of issuing tickets. Must the student have a certificate as evidence of examination?

*Prof. Abbott*—I move that all students shall have a certificate from the school he was examined in, which certificate shall be required by other colleges before they admit him.

*Prof. Hunt*—Suggested that the Executive Committee prepare blank certificates, to be filled up by such subjects as he has been examined in, and a plain statement as to what the examination has been.

*Prof. Darby*—This seems superfluous, as the certificate would always be demanded; so the matter is well able to take care of itself.

*Prof. Hunt*—I still insist on some uniformity in the matter.

*Prof. Abbott*—Said he thought the subject should be referred to a committee, to report next year.

*Prof. Smith*—Agreed with Prof. Darby that the thing was superfluous.

Prof. Abbott's resolution was then carried unanimously.

*Prof. Taft*—Chairman of the Committee on Curriculum, submitted the accompanying report:

### CURRICULUM.

We recommend that the following subjects and arrangements be adopted by the colleges of this association, viz:

1st year—Anatomy, with dissections; physiology, histology, chemistry, didactic and practical; mechanical dentistry.

2d year—Review of the junior year studies, pathology, surgery, materia medica, therapeutics and operative dentistry.

*Prof. Truman*—Moved that "review of junior year studies" be stricken out.

No second.

*Prof. Abbott*—Moved the adoption of the report as a recommendation. Carried unanimously.

*Prof. Foster*—Moved that a committee be appointed to confer with the National State Board of Dental Examiners, to let them know what has been done by this association.

*Prof. Taft*—Said that this was feasible, and he had no doubt at all but that that association will recognize the action of this association, and he thought it could not do otherwise than discriminate between the colleges of the association and other colleges.

The Chair remarked that this committee must be furnished with attested copies of the resolutions passed by this body.

The resolution was then unanimously adopted.

The Chair appointed on this committee Profs. Foster, Abbott and Hunt.

*Prof. Truman*—Moved that the Secretary be authorized to prepare printed copies of all the resolutions passed, and that they be sent to all schools represented in the association.

*Prof. Hunt*—Suggested that the official results of the meeting also be included upon the slips.

The motion was carried unanimously.

The association then adjourned, to meet again in one year, or as the Executive Committee might designate.

---

### MODELING COMPOUND.

The following formula will make a compound quite equal to any in the market, while its expense is comparatively little. Its consistency may be varied by changing the proportions of the ingredients.

| | |
|---|---|
| Gum Cowry, | $\frac{3}{4}$ lb. |
| Stearine, | 1 " |
| French Chalk, | $1\frac{1}{2}$ " |

Melt the Stearine in a zinc pan, add the Gum Cowry and mix thoroughly. Lastly, stir in the powdered chalk.

